# Unoccupied Beds in Cottage Hospitals

**Published:** 1923-09

**Authors:** Chas. E. S. Flemming

**Affiliations:** Manvers House, Bradford-on-Avon


### CORRESPONDENCE.

[Correspondence on all subjects is invited, but we cannot be responsible for the opinions expressed by our correspondents, who must give name and address as a guarantee of good faith, but not necessarily for publication. Correspondents are reminded that conciseness greatly facilitates early insertion.]

### Unoccupied Beds in Cottage Hospitals.

*To the Editor of* The Hospital and Health Review.

Sir,—Hospitals are clamouring for more beds, and they have long waiting lists, but out of the 29,396 beds in the large hospitals, 6,013 were vacant throughout the whole of the year 1922. This statement, of course, will not stand criticism, but it is founded on the figures given on page 13 of the Report on the Voluntary Hospitals in 1922 and is phrased in the manner of the statement made about Cottage Hospitals—that the Cottage Hospital idea may have been rather overdone because of the steady falling off in the number of occupied beds, and that in England and Wales, excluding London, they had 1,839 beds vacant throughout the whole of last year.

No one appreciates more than myself the value of Sir Napier Burnett's report, but I regret that he has lent the weight of his great authority to this statement. I feel quite sure that he did not mean it to be interpreted as I have done, but I am afraid that some people reading it will take it in that sense. Of course, those who are acquainted with hospital statistics will realise that these Cottage Hospital beds were not unoccupied, in the sense that they were otherwise available "all the year round." I am glad that attention has been drawn to the fact that the fullest use is not made of all the beds in many Cottage Hospitals, and this is a fact that requires, as Sir Napier justly says, very serious consideration.

The figure he gives requires careful analysis. The average of unoccupied Cottage Hospital beds—1,839—is, no doubt, too high, but it will be admitted that the proportion of unoccupied beds in any hospital is necessarily greater the smaller the number available. In group A it is nearly 18 per cent., in group B 27 per cent. Now, if we take the latter percentage and apply it to group C, Cottage Hospitals, we find that they have 1,234 beds unavoidably vacant during the year. That leaves 605 what one may call avoidably unoccupied, that is only about two per hospital. Sir Napier suggests that these vacant beds should be used for convalescent cases from the Central Hospitals and for maternity cases. One of the practical difficulties in adopting his suggestion is that these beds are empty for irregular periods and at indefinite times. As regards convalescent cases, I was of his way of thinking until in this county two years ago we drafted a scheme for the better co-ordination and improvement of the hospital service. It was then found, for various reasons, to be quite impracticable to make use of Cottage Hospital beds for convalescents from Central Hospitals unless extra beds could be specially provided for the purpose. As for maternity cases, I hope the day may come when every Cottage Hospital will have accommodation for these patients, but I am sure that it would not be possible or desirable to make use for this purpose of the beds in a general ward.

Sir Napier has drawn attention to the fact that there are some hospitals that do not do enough and others that at times attempt too much; but of course it is peculiar to Cottage Hospitals that their activities must depend upon the practitioners who happen to serve them, so that there must always be considerable difference in the quantity and the nature of the work they do. The Cottage Hospital should provide for those cases that can be treated by the general practitioners of the neighbourhood and do not require the special skill and equipment found in the large hospitals, where at present many beds are occupied by patients who might quite well be treated in the surrounding general practitioner hospitals.

I have written the above fearful lest Sir Napier's report should inadvertently check the growth of this important part of the medical service of the country. There is no doubt that in many instances these institutions require reorganising and reforming, but that is no reason for reducing their number or their size. In fact, experience has shown that an increase in the number of beds in a Cottage Hospital often means a higher average of occupation.

Chas. E. S. Flemming.

Manvers House, Bradford-on-Avon.

